# Review of Signaling Pathways Governing MSC Osteogenic and Adipogenic Differentiation

**DOI:** 10.1155/2013/684736

**Published:** 2013-12-12

**Authors:** Aaron W. James

**Affiliations:** Department of Pathology & Laboratory Medicine, David Geffen School of Medicine, University of California, Los Angeles, 10833 Le Conte Avenue, CHS A3-251, Los Angeles, CA 90077, USA

## Abstract

Mesenchymal stem cells (MSC) are multipotent cells, functioning as precursors to a variety of cell types including adipocytes, osteoblasts, and chondrocytes. Between osteogenic and adipogenic lineage commitment and differentiation, a theoretical inverse relationship exists, such that differentiation towards an osteoblast phenotype occurs at the expense of an adipocytic phenotype. This balance is regulated by numerous, intersecting signaling pathways that converge on the regulation of two main transcription factors: peroxisome proliferator-activated receptor-**γ** (PPAR**γ**) and Runt-related transcription factor 2 (Runx2). These two transcription factors, PPAR**γ** and Runx2, are generally regarded as the master regulators of adipogenesis and osteogenesis. This review will summarize signaling pathways that govern MSC fate towards osteogenic or adipocytic differentiation. A number of signaling pathways follow the inverse balance between osteogenic and adipogenic differentiation and are generally proosteogenic/antiadipogenic stimuli. These include **β**-catenin dependent Wnt signaling, Hedgehog signaling, and NELL-1 signaling. However, other signaling pathways exhibit more context-dependent effects on adipogenic and osteogenic differentiation. These include bone morphogenic protein (BMP) signaling and insulin growth factor (IGF) signaling, which display both proosteogenic and proadipogenic effects. In summary, understanding those factors that govern osteogenic versus adipogenic MSC differentiation has significant implications in diverse areas of human health, from obesity to osteoporosis to regenerative medicine.

## 1. Introduction

**Figure 1:**
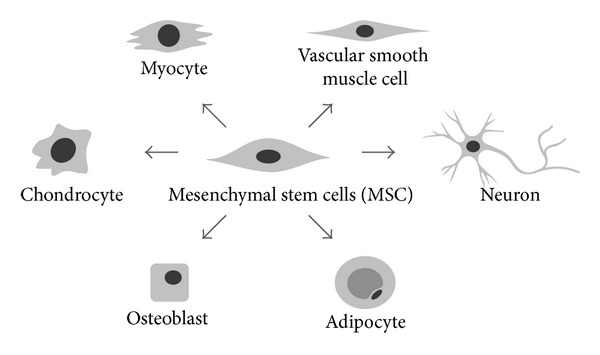
Multilineage differentiation of mesenchymal stem/stromal cells (MSC). Multipotent mesenchymal stem cells (MSC) have been derived from numerous vascularized tissue sources, including bone marrow, adipose, and skeletal muscle tissue, among others. Multilineage differentiation includes osteoblastic, chondrogenic, myogenic, smooth muscle, and neurogenic differentiation. With progressive differentiation toward a mature cell phenotype, often the capacity for differentiation down a competing lineage is lost.

Mesenchymal stem cells (MSC) are multipotent stromal cells capable of self-renewal and capable of multilineage mesenchymal differentiation [1]. These nonhematopoietic cells can differentiate down multiple mesenchymal lineages, including osteogenic, chondrogenic, adipogenic, myogenic, and neurogenic lineages [2] (Figure 1). Originally identified in the bone marrow, MSC are readily obtained from numerous mesenchymal tissue types, including skeletal muscle and adipose depots. In particular, adipose tissue is an attractive source for MSC isolation, as it is readily accessible with minimal morbidity by routine liposuction procedures [3–5]. Indeed, human adipose-derived stromal cells (or hASC) have been demonstrated to have significant potential for use in tissue engineering applications, as shown in preclinical animal models [6]. However, the uncultured stromal vascular fraction of adipose tissue represents a heterogeneous cell population that is not immediately suitable for bone formation, prompting investigators to search for alternative methods for MSC purification other than culture propagation [5, 7]. Alternative sources for MSC derivation include nearly any vascularized tissue, from umbilical cord to oral gingiva [8, 9]. Indeed, the perivascular origin of MSC has become an increasingly accepted theory [10–13].

MSC derived from bone marrow (BMSC) are relatively scarce in number but like all MSC have a capacity for repeated culture expansion while retaining their growth potential and multipotency [2]. BMSC typically express cell markers such as CD29, CD44, CD73, CD105, and CD166 and are negative for hematopoietic markers [2, 14]. However, it is worth noting that, with the diversity in sources and protocols for derivation, MSC cell identity remains relatively poorly defined across species, tissue type, and culture strain [15]. Upon induction and differentiation towards a specific mesenchymal lineage, the gene expression of MSC shifts until the phenotype is characteristic of the target cell. While MSC differentiation can be directed by multiple microenvironmental factors (such as mechanical forces [16], electrical currents [17–19], and magnetic fields [20]), this review will specifically focus on cytokine signaling that govern MSC lineage differentiation.

As mentioned, MSC function as precursors to a variety of mature mesenchymal cell types, including adipocytes. Various theoretical definitions of the process of adipocyte differentiation, or adipogenesis, have been put forth. Sinal and colleagues characterize adipogenesis in two phases: the determination phase and the terminal differentiation phase [21]. During the determination phase, multipotent MSC commit to the adipocyte lineage. Morphologically, preadipocytes have a fibroblastic phenotype and are not readily distinguishable from their MSC precursors. During the terminal differentiation phase, preadipocytes become adipocytes and acquire new functions, including lipid synthesis and storage, as well as adipocyte-specific protein production [22]. Rosen and colleagues define adipogenesis as a shift in gene expression from MSC to a phenotype that defines mature adipocytes [23], including expression of CD24, CD29, CD34, and CD36, among others [24–26]. Overall, adipogenesis is a sequentially and temporally ordered process involving multiple signaling cascades that converge at the level of peroxisome proliferator-activated receptor-*γ* (PPAR*γ*) transcriptional activity [21, 23].

Of course, MSC also give rise to osteoblasts to form bone [2]. The process starts with commitment of osteoprogenitor cells and differentiation into pre-osteoblasts, which eventually develop into mature osteoblasts [27]. In turn, mature osteoblasts will become entombed in osteoid to become osteocytes. At its most basic level, osteoblast differentiation requires expression of the key transcription factor, Runt-related transcription factor 2 (Runx2) [27], which will be reviewed in the coming sections. However, Runx2 expression is not sufficient for osteoblast maturation, as other transcriptions factors and extracellular signals reviewed in this chapter are also involved [28]. The development of an immature osteoblast into a mature one can be categorized into phases of proliferation, maturation, matrix synthesis, and matrix mineralization (reviewed in [27]). Osteoblasts synthesize bone matrix to initially form bone and later function in bone remodeling and mineral metabolism [28].

**Figure 2:**
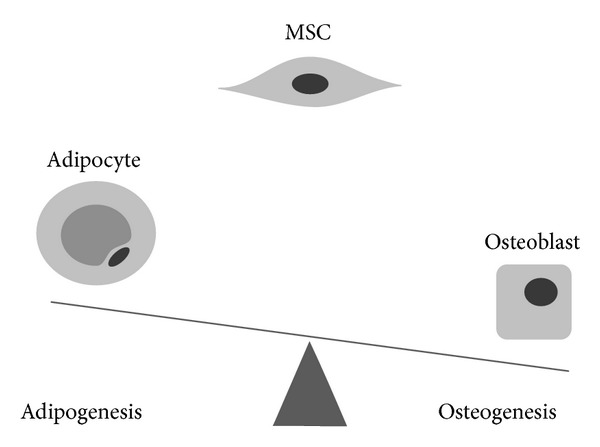
Theoretical inverse relationship between osteogenic and adipogenic programming. Multiple signaling pathways have been demonstrated to preferentially induce osteogenic programming at the expense of adipogenesis, or vice versa. In this regard, the differentiation of an MSC into either an adipocytic or osteoblastic phenotype can be theorized as a seesaw, where induction of one lineage comes at the expense of the other. However, numerous exceptions exist to this simplification.

The commitment and differentiation of MSC towards an adipogenic or osteogenic cell fate depend on a variety of signaling and transcription factors. A large body of experimental evidence suggests that an inverse correlation exists between adipogenesis and osteogenesis (Figure 2) [29, 30]. The evidence for an inverse relationship is primarily based on *in vitro* studies in which culture supplements upregulate osteogenic differentiation with associated downregulation of adipogenic differentiation, or vice versa [31–34]. Several bipotent or multipotent cell lines are commonly used. These include the pluripotent C3H10T1/2 cell line and the murine BMSC line M2-10B4 [35, 36]. Several cell signaling cascades exemplify proosteogenic/antiadipocytic stimuli and will be discussed below. These include *β*-catenin dependent Wnt signaling (as well as *β*-catenin independent signaling) [37, 38], Hedgehog signaling [39, 40], and NELL-1 (NEL-like protein 1) signaling [41, 42]. Dissimilarly, various signaling cascades demonstrate positive regulation of both osteogenesis and adipogenesis. Perhaps the most clinically relevant examples are bone morphogenetic proteins (BMPs), of which BMP-2 and BMP-7 are available for orthopaedic application [43, 44]. While the majority of BMPs promotes osteogenic commitment and differentiation of MSC [45, 46], BMPs also demonstrate proadipogenic effects [47, 48]. Insulin-like growth factor (IGF) signaling likewise demonstrates dual proosteogenic/proadipogenic effects. This review will sequentially discuss the effects of these diverse signaling cascades that coordinately govern MSC osteogenesis and adipogenesis.

## 2. Control of Adipogenesis and Osteogenesis by Transcription Factor Activity: Runx2 and PPAR*γ*

Signaling cascades which promote MSC osteogenic and/or adipogenic lineage differentiation generally converge on two key transcription factors: PPAR*γ* and Runx2. PPAR*γ* is generally considered the master regulator of adipogenesis and also has well-described anti-osteoblastogenic effects. Likewise, Runx2 is regarded as the master regulator of osteogenesis. Together, they are in large part responsible for mediating the effects of various cytokines in determination of adipogenic versus osteogenic MSC differentiation. Typically, increased expression of one transcription factor is associated with downregulation of the other [49–52]. Of course, a number of other key transcriptional factors exert effects independent and in association with Runx2 and PPAR*γ*. For example, Osterix and CCAAT/enhancer-binding family of proteins (C/EBP) play important adjunctive roles (see [53, 54] for a comprehensive review of the osteogenic and adipogenic functions of Osterix and C/EBP).

## 3. The Master Osteogenic Transcription Factor, Runx2

Originally identified as the binding site for polyomavirus enhancer binding protein (PEBP), Runx was later identified as the Moloney murine leukemia virus enhancer core binding protein [55]. The Runx family consists of three distinct proteins: Runx1-3, all of which are comprised of a varying *α* subunit with the same *β* subunit [56, 57]. In order to bind to DNA, Runx proteins must form a heterodimer with transcriptional coactivator core binding factor *β* (Cbf*β*), a cotranscription factor [56]. The DNA binding domain of the Runx family, known as *Runt, *is homologous to the *Runt *sequence in Drosophila. Members of the Runx family have various roles in determining stem cell commitment; Runx1 determines hematopoietic stem cell differentiation [58], Runx2 determines osteoblastic and chondrogenic cell differentiation [59], and Runx3 has roles in epithelial differentiation, neurogenesis, and chondrocyte differentiation [60, 61]. Runx has also been postulated as both an oncogene and tumor suppressor: Runx family loss of function seems to be a key event in certain myeloid, lymphoid, and epithelial cancers [62, 63]. Retroviral overexpression of Runx2 has demonstrated oncogenic functions [64]. However, data does suggest that Runx3 acts as a tumor suppressor, as it is methylated and downregulated in cancer derived cell lines [65–68]. As the Runx family is structurally similar, it is possible that tissue-specific Runx activity allows for its complex role in carcinogenesis. In regard to osteogenic differentiation, Runx2 activates and regulates osteogenesis as the targeted gene of many signaling pathways, including but not limited to transforming growth factor-beta 1 (TGF-*β*1), BMP, Wingless type (Wnt), Hedgehog (HH), and (Nel)-like protein type 1 (NELL-1) [69–71]. Mice with a homozygous mutation for Cbfa-1 deficiency (*Runx2* ^−/−^) have an absence of differentiated osteoblasts and bone and die shortly after birth [72]. Such *Runx2* null phenotypes cannot be rescued by the overexpression of other osteogenic factors, although the cleidocranial dysplasia-like phenotype of *Runx2* ^+/−^ mice can be partially rescued [73, 74]. While Runx2 is not a key regulator of adipocyte differentiation, its function in promoting osteogenesis may subvert potential adipocyte lineage differentiation in MSC.

## 4. The Master Adipogenic Transcription Factor, PPAR*γ*

Peroxisome proliferator-activated receptors are members of the steroid/thyroid hormone receptor gene superfamily [75]. Initially named for PPAR*α* [76], subsequent structural analogs PPAR*δ* and PPAR*γ* were since discovered. All three PPARs are found in mammals and are activated by polyunsaturated fatty acids [77], interacting with binding sites on targeted genes by forming heterodimers with the retinoid X receptor (RXR) in order to recruit transcriptional coactivator proteins [78]. While both PPAR*α* and PPAR*δ* are expressed during adipogenesis, PPAR*γ* is adipocyte restricted and more rapidly increases in expression during early adipogenesis [79, 80]. PPAR*γ* is expressed during adipogenesis as two isoforms, PPAR*γ*1 and PPAR*γ*2, the latter being predominant in adipose tissue [21]. PPAR*γ*1 is expressed at lower levels in adipose tissue among other tissues, including breast and prostatic tissue [81–83]. PPAR*γ* is principally regarded as the master regulator of adipogenesis, for no other factor can rescue adipocyte formation in the event of PPAR*γ* knockout, and generally all proadipogenic cell signaling pathways converge with PPAR*γ* [84].

It is currently believed that a ligand-dependent activation of PPAR*γ* must occur for any proadipogenic effects. Even then, the ligand is only necessary in the commitment phase for the adipocyte lineage, whereas PPAR*γ* expression is necessary for both commitment and differentiation phases [84, 85]. One study demonstrated that differentiation of non-adipogenic fibroblasts required PPAR*γ* activation through exposure to an exogenous ligand. By contrast, preadipocytes were able to continue with adipogenic differentiation without exposure to ligand [84]. One such set of ligands for PPAR*γ* is thiazolidinediones (TZDs), which are potent PPAR*γ* agonist among several other derivatives of polyunsaturated acids [86]. Recently, there have been several endogenous molecules derived from fatty acids found to bind and activate PPAR*γ*, although induced adipogenesis [84, 85]. Moreover, recent studies show that ectopic expression of a mutant form of PPAR*γ* without functional ligand-binding domains was able to support adipocyte differentiation [87], which inserts some doubt into the absolute requirement for PPAR*γ* ligand activation.

Studies from genetic manipulation of PPAR*γ* in mice have confirmed its central role in adipogenic differentiation. Cells derived from PPAR*γ* ^+/−^ mice demonstrate a reduced ability to differentiate into adipocytes [84]. PPAR*γ*-deficient embryonic stem cells fail to differentiate into adipocytes and instead differentiate into osteoblasts. Additionally, PPAR*γ* ^+/−^ mice have demonstrated increased bone mass with increased osteoblastogenesis, while having a marked decrease in fat stores [84]. Similarly, mice with mutation in PPAR*γ*2 have decreased expression of both PPAR*γ*1 and PPAR*γ*2 in white adipose tissue, while exhibiting increased bone formation [47]. In another approach, selective deletion of PPAR*γ* in murine adipose tissue led to a loss of both brown and white adipocytes [22].

There is much evidence supporting the anti-osteoblastogenic and proadipogenic properties of PPAR*γ*. Several PPAR*γ* agonists/ligands, namely, TZD rosiglitazone and 15-deoxy-delta (12,14)-PGJ_2_, promote BMSC adipogenesis while inhibiting osteogenesis [88, 89]. However, not all agonists obtain this effect, as it depends on affinity of the ligand. For example, the partial agonist GW0072 inhibits MSC osteogenesis without necessarily affecting adipogenesis. In contrast, 9-hydroxyoctadecadienoic acid stimulates adipogenesis while not affecting osteoblastogenesis [88]. A similar pattern is seen *in vivo, *where chronic treatment of mice with low-affinity TZD troglitazone induces increased bone marrow adipocytes, without affecting bone mass [90]. Conversely, treatment with high-affinity TZD rosiglitazone decreases bone mineral density, rate of bone formation, and trabecular bone volume in addition to upregulating bone marrow adiposity [90, 91]. This inhibition of osteogenesis by high-affinity rosiglitazone was also associated with suppression of osteogenic transcription factors, including Runx2 [89]. Low-affinity agonist, netoglitazone, weakly inhibited osteoblastogenesis while inducing adipogenesis *in vitro* in a PPAR*γ*2-dependent manner [89]. *In vivo*, neglitazone did not demonstrate an effect on bone, with unaffected expression levels of Runx2 [89].

## 5. Control of Adipogenesis and Osteogenesis by Wnt Signaling

**Figure 3:**
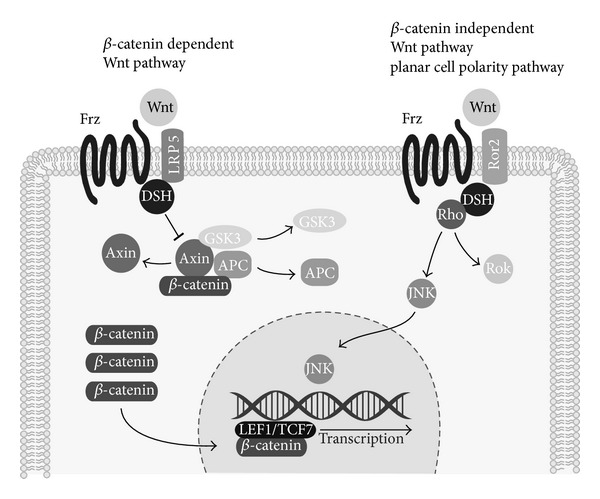
Schematic of *β*-catenin dependent and independent Wnt signaling pathways. Wnt signaling transduction occurs via *β*-catenin dependent or *β*-catenin independent signaling pathways. In *β*-catenin dependent signaling, extracellular Wnt ligands bind to the LRP5-Frizzled (Frz) complex to activate intracellular disheveled (DSH). This subsequently inhibits the intracellular complex comprised of axin, glycogen synthase kinase 3 (GSK3), and adenomatosis polyposis coli (APC) protein. This inhibits the cytosolic degradation of *β*-catenin, which accumulates and is free to enter the nucleus to heterodimerize with lymphoid enhancer-binding factor/T cell factor (LEF/TCF1) and mediate effects of gene transcription. Under the *β*-catenin independent signaling pathway, a similar transmembrane complex forms between Wnt, Frz, DSH, and Ror2 and activates secondary messengers.

Over the course of the past several decades, wingless-type MMTV integration site (Wnt) signaling has been identified to play an essential role in cell fate determination, proliferation, and differentiation [92, 93]. Dysregulation/hyperactivation of Wnt signaling is associated with numerous diseases such as neurodegeneration [94], gastrointestinal cancers [95], and osteoporosis [92]. To date, over nineteen Wnt receptors and coreceptors have been identified throughout seven families of proteins [93]. Collectively, Wnt signaling has demonstrated both proosteogenic and antiadipogenic activities, through both canonical (*β*-catenin dependent) and noncanonical (*β*-catenin independent) pathways (Figure 3).

The *β*-catenin dependent pathway initiates with the binding of extracellular Wnt ligands to the seven-pass transmembrane frizzled receptors (Frz) expressed at the cell surface [96]. This induces complex formation with transmembrane low-density lipoprotein receptor (LRP5/6) coreceptor, as well as intracellular proteins of the disheveled (DSH) family [97]. The resulting activation of DSH then functions to inhibit a second, intracellular complex comprised of axin, glycogen synthase kinase 3 (GSK3), and adenomatosis polyposis coli (APC) protein (Figure 3). GSK3 normally phosphorylates *β*-catenin, promoting its degradation. Wnt stimulation inhibits the Axin/GSK3/APC complex, and *β*-catenin accumulates rather than being degraded, and levels of nuclear *β*-catenin increase. Once inside the nucleus, *β*-catenin can heterodimerize with lymphoid enhancer-binding factor/T cell factor [97]. Ultimately, *β*-catenin dependent Wnt signaling elicits gene transcriptional activity to influence MSC lineage determination [98] (see [92] for a more comprehensive review). While the noncanonical Wnt pathway is similar in that it involves extracellular Wnt binding to frizzled receptors (Frz) and DSH downstream, it otherwise diverges to mediate its effects through a *β*-catenin independent manner [99–101]. Please see [102] for a more detailed review of noncanonical Wnt signaling.

Canonical Wnt signaling has well-established effects on bone mass in both animal models and human patients. LRP5 mutational studies first identified a critical role for Wnt signaling in bone maintenance [103]. LRP5 loss-of-function mutations cause pseudo-glioma syndrome, characterized by a low bone mass phenotype. Conversely, LRP5 gain-of-function mutations result in a high bone mass phenotype [104–106]. A direct role for *β*-catenin in regulating osteoblast and osteoclast activity has been repeatedly observed [107]. For example, in mesenchymal osteoblastic precursors, *β*-catenin deficiency leads to arrest of osteoblast development at an early stage and consequent embryonic skeletal defects [107–110]. Similarly, in committed osteoblasts, *β*-catenin deficiency results in impaired maturation and mineralization [111, 112]. As well, Wnt/*β*-catenin signaling activity in both mature and osteoblastic precursors leads to altered OPG/RANKL elaboration and secondary reductions in osteoclast activity and bone resorption [113, 114]. Accordingly, current clinical applications for osteoporosis target Wnt inhibitors to stimulate formation of new bone and inhibit bone resorption, or so-called “inhibitors to Wnt inhibitors.” Currently targeted Wnt signaling antagonists include Sclerostin (SOST) and Dickkopf-1 (DKK1) [115]. Expectedly, inhibition of these antagonists, via anti-SOST and anti-DKK1, respectively, has been shown to stimulate bone formation and increase bone mineral density, with phase II clinical trials (for anti-SOST) and preclinical trials (for anti-DKK1) underway [116–118]

Various members of the Wnt signaling family have been identified to inhibit the early stages of adipogenesis [119]. For example, WNT10B has been shown to maintain 3T3-L1 preadipocytes in an undifferentiated state via inhibition of PPAR*γ* and C/EBP-*α* [120–122]. Similarly, activation of *β*-catenin via ectopic expression of Wnt1 also leads to direct suppression of PPAR*γ* and prevention of 3T3-L1 cell adipogenic differentiation [120, 121]. Interestingly, this negative inhibition is reciprocal, in that upregulation of PPAR*γ* functions to inhibit *β*-catenin signaling [120, 121, 123]. Conversely, inhibition of Wnt/*β*-catenin signaling via treatment with DKK family proteins positively regulates adipogenesis [119, 120, 124]. Further studies suggest that the canonical ligand Wnt3a, among several others, inhibits activation of both PPAR*γ* and C/EBP*α* in order to elicit its antiadipogenic effects [125]. However, while PPAR*γ* upregulation may negatively regulate Wnt/*β*-catenin signaling, overexpression of PPAR*γ* and/or C/EBP*α* is not sufficient in rescuing Wnt/*β*-catenin-mediated inhibition of adipogenesis [21, 125].

In general, Wnt/*β*-catenin signaling pathway activation follows the inverse pattern between the induction of MSC osteogenic and adipogenic differentiation. The activation of Wnt/*β*-catenin, via lithium chloride, for instance, inhibits GSK3b, which results in general in both the promotion osteogenesis and the suppression of adipogenesis [126, 127]. Similarly, Wnt10b stimulates osteogenesis *in vivo* to increase bone mass while blocking adipogenesis in preadipocytes *in vitro* via stabilization of free cystolic *β*-catenin [120, 124, 128]. Other canonical Wnt ligands, such as Wnt6 and Wnt10a, exhibit similar effects in stimulating osteogenesis while also inhibiting adipogenesis [129]. Not surprisingly, disruption of Wnt/*β*-catenin impairs osteogenesis *in vitro *[111, 112] while increasing adipogenesis both *in vitro* and *in vivo* [120, 124, 130]. Moreover, inhibitors of the Wnt/*β*-catenin pathway also demonstrate consistency with this inverse relationship between osteo- and adipogenic differentiation. DKK1, for instance, which is secreted by preadipocyte cells, inhibits osteogenesis while promoting adipogenesis *in vitro* [131]. The inverse relationship carries over to the noncanonical branch of Wnt signaling as well. Wnt5a, for instance, has been shown to suppress proadipogenic PPAR*γ* transactivation when coinduced with proosteogenic Runx2 in MSC [21, 132]. Thus, seen across multiple ligands and inhibitors, Wnt signaling generally exerts proosteogenic and antiadipogenic effects in both canonical or noncanonical signal transduction pathways.

## 6. Control of Adipogenesis and Osteogenesis by Hedgehog Signaling

Since its original discovery in Drosophila, the Hedgehog (HH) protein family has been identified in all vertebrates and classified into three structural homologues: Sonic Hedgehog (SHH), Indian Hedgehog (IHH), and Desert Hedgehog (DHH). DHH expression is typically limited to male reproductive tract [133] and will not be further discussed. SHH and IHH are critical during embryological development. In particular, SHH plays a key role during skeletogenesis, involved in patterning of the axial, appendicular, and facial skeleton [134, 135]. Closely related to SHH through gene duplication, IHH regulates both chondrogenesis and endochondral bone formation [136]. In fact, disruption of HH signaling results in severe skeletal abnormalities, the most common of which is holoprosencephaly [137]. In regulation of stem cells, SHH is a critical moderator of cell differentiation, as it demonstrates proosteogenic and antiadipogenic properties in multiple MSC types [39].

**Figure 4:**
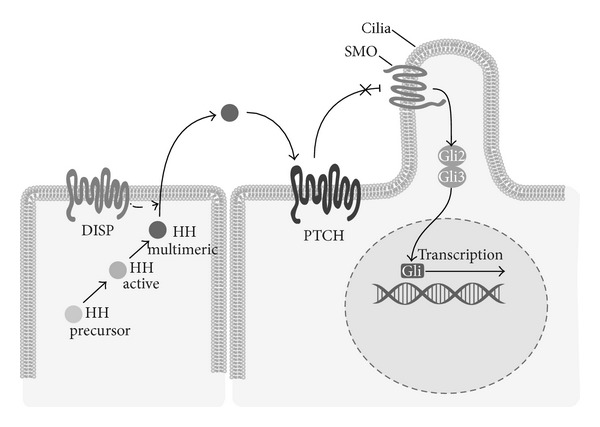
Schematic of Hedgehog signaling pathway. The initially insoluble Hedgehog (HH) ligand precursor undergoes a series of intracellular modifications before reaching an active, multimeric form. Following release from the membrane by Dispatched (DISP), the morphogen binds to Patched (PTCH), which releases Smoothened (SMO) from constitutive inhibition by PTCH. This activates the Gli2/3 complex, which goes on to promote gene expression of Gli1, while repressing the transcriptional repressor Gli3.

All three HH morphogens follow the same, highly conserved HH signaling pathway (Figure 4). First, the insoluble HH polypeptide precursor undergoes conversion into a soluble, multimeric form capable of diffusing across the cell membrane. This is then autocatalytically processed from a 45 kD to a 19 kD protein, with modifications for a cholesterol moiety at the C-terminal and palmitate at the N-terminal [138]. Subsequently, the modified HH morphogen is secreted from the cell via Dispatched, a large transmembrane protein, after which it binds to the receptor Patched (PTCH), a 12-pass transmembrane protein, on the receiving cell. This binding to PTCH relinquishes Smoothened (SMO), a 7-pass transmembrane protein, from PTCH suppression, thereby enabling activation of the glioblastoma gene products (Gli) family of transcription factors (Gli1-3). Since Gli1 is a target gene of the HH pathway, it is used as a reliable marker for HH signaling activity [84]. It is important to note that HH signal transduction occurs at the primary cilia and that intraflagellar transport (IFT) proteins are required to preserve cilia during HH signaling [135]. Accordingly, these IFT proteins are essential in transferring transmembrane proteins PTCH and SMO, as movement through the cilium is required to upregulate genes targeted by HH signaling [84]. While being not fully understood, it is currently believed that HH signal transduction is mainly mediated though the Gli transcription factors, and that they are responsible for HH-induced lineage commitment during MSC differentiation.

The antiadipogenic potential of HH signaling in MSC has been observed across a variety of adipocyte and multipotent cell lineages. Generally, adipogenesis in MSC, as it relates to HH signaling, occurs as a result of decreased Gli1, Gli2, Gli3, and PTCH expression [40]. Conversely, when the HH pathway is upregulated via SMO-activated inducer of HH signaling, such as purmorphamine [139], there is a significant decrease in adipocyte-specific markers: adipocyte fatty acid binding protein, adipsin, CD36, adiponectin, and leptin. Through the inhibition of adipogenic genes, HH signaling ultimately decreases sensitivity to insulin, which in turn reduces the expression of adipogenic transcription factors, C/EBP*α* and PPAR*γ* [40]. Moreover, *in vitro* studies evaluating RNAi scans on Drosophila genome have confirmed the antiadipogenic function of HH signaling. Specifically, HH signaling blocked differentiation of white adipocytes. Likewise, transgenic activation of HH signaling in both Drosophila and mammalian models impaired fat formation [140, 141]. Using multipotent C3H10T1/2 cells, treatment with SHH resulted in the suppression of the proadipogenic effects of bone morphogenetic protein (BMP)2 [142].

In addition to its antiadipogenic properties, HH signaling is well known to stimulate MSC osteogenic differentiation. While the exact mechanism and stage at which HH acts during osteoblastogenesis are not completely understood, both *in vivo* and *in vitro* data suggest that bone formation occurs via a positive feedback loop. That is, HH-induced osteoblastogenesis requires BMP signaling, and together they elicit a synergistic expression of alkaline phosphatase activity [143]. This positive feedback loop is further mediated by Gli2 transcription, which serves to upregulate BMP-2 expression, which in turn activates Gli transcription [144]. In the murine MSC line C3H10T1/2, HH simultaneously induced osteoblastic differentiation while inhibiting adipogenesis [145–147]. In KS483 cells, a similar induction of osteogenesis via SHH was observed alongside inhibited adipogenesis, despite adipogenic culture conditions [148]. It is important to note that SHH induced differentiation was only observed in immature mesenchymal cell lines 3H10T1/2 and not pre-osteoblastic MC3T3-E1 or osteoblastic cell lines OS 17/2.8 and ROB-C26 [143, 147]. These data imply that SHH activity may be key in stimulating osteoblastogenesis only during early stages of cell differentiation. In summary, current data suggest that HH signaling promotes MSC osteogenic differentiation over adipogenic differentiation, primarily via Gli transcriptional factor activity.

## 7. Control of Adipogenesis and Osteogenesis by NELL-1 Signaling

The secreted molecule NELL-1 (NEL-like protein 1) was first discovered to have osteoinductive properties by its overexpression during premature bone formation in human sporadic coronal craniosynostosis [149, 150]. NELL-1 is expressed during both intramembranous and endochondral bone formation. Overexpression increases both differentiation and mineralization selectively in osteoblasts and is highly specific to the osteochondral lineage [151]. Transgenic mice overexpressing NELL-1 show premature cranial suture fusion and bone overgrowth, thus replicating the human observed phenotype [152]. Interestingly, the nontissue specific overexpression of NELL-1 in mice only manifested phenotypes in the calvarial bone. This finding suggests a relative osteo-specific effect of NELL-1 signaling. Conversely, downregulation of NELL-1 resulted in inhibited osteoblastogenesis *in vitro* in primary cultures of fetal rat calvarial cells and MC3T3 cell line cultures [152]. Moreover, complete loss of NELL-1 in mice results in significant reduction in the mineralization of calvarial bones and attenuated osteoblastogenesis [153]. Thus, NELL-1 has been shown to have a critical role in craniofacial osteogenic differentiation and bone formation [152].

The osteoblastogenic effects of NELL-1 have been studied in the context of bone tissue engineering. For example, *in vivo* NELL-1 administration induces significant calvarial defect healing in rats [154]. When NELL-1 was applied to a PLGA scaffold in a rat calvarial defect, decreased Osterix-producing cells were observed, concomitantly with increased bone sialoprotein, osteocalcin, and BMP-7 [149]. *In vivo*, several studies have demonstrated that NELL-1 has comparable bone regeneration capacity as BMP-2, in both calvarial defect and spinal fusion models, among others [149, 155]. NELL-1 has also been applied to critical-sized femoral segment defect models in rats, observing to enhanced bone regeneration/osseous union [156]. A variety of spinal fusion models have also been investigated across several animal models. For example, NELL-1 demonstrated osteoinductive properties in rat spinal fusions [154, 157], using apatite coated alginate/chitosan microparticles and *β*-TCP scaffolds [158]. In a sheep spinal fusion model using demineralized bone graft, NELL-1 increased both bone volume and mineral density at three months, with a similar bone-forming efficacy to BMP-2 [155]. Overall, NELL-1 demonstrates robust induction of bone throughout many *in vivo* models, ranging from rodents to large preclinical animals [151].

**Figure 5:**
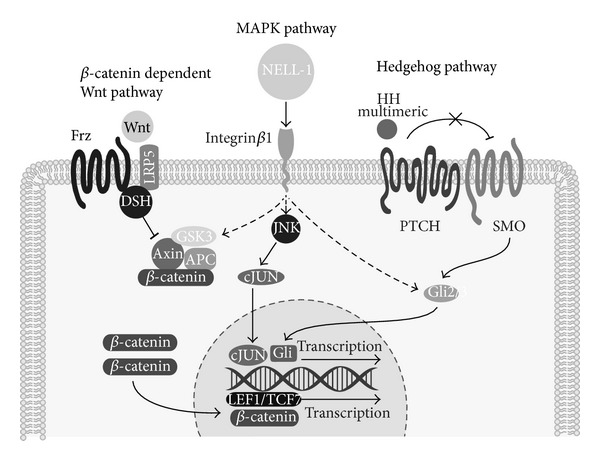
Schematic of NELL-1 signaling pathway. NELL-1 is a secreted osteoinductive protein that binds to the cell surface receptor Integrin*β*1. Binding to Integrin*α*3 has also been reported. Multiple intracellular signaling pathways have been shown to increase after NELL-1 stimulation, including MAPK, Hedgehog, and *β*-catenin dependent Wnt signaling. Although the relative importance of these pathways is still undefined, NELL-1 treatment results in increased *Runx2* transcription, Runx2 phosphorylation, and induction of osteogenic programming.

Mechanistically, NELL-1 is directly regulated by the transcription factor Runx2 [74, 151, 154]. NELL-1 is preferentially expressed in osteoblasts in levels similar to Runx2 and is most highly expressed during skeletogenesis [74, 151]. In *Runx2* deficient mice, overexpression of NELL-1 was not sufficient to rescue mineralization, whereas absence of NELL-1 significantly decreased Runx2 activity *in vitro* [74]. Integrin*β*1 was recently identified as the first cell surface receptor of NELL-1 [159]. Cell surface binding in a pre-osteoblast cell line required Integrin*β*1 expression [159]. Moreover, siRNA for Integrin*β*1 blocked at least some of the cellular effects of NELL-1, including induction of pre-osteoblast attachment [159]. NELL-1 is known to promote osteogenesis accompanied by activation of MAPK, canonical Wnt and HH signaling [41, 42, 160, 161] (Figure 5). NELL-1 activates both ERK1/2 and JNK1 MAPK pathways in Saos-2 osteosarcoma cell type [160]. This activation of MAPK signaling is associated with Runx2 protein phosphorylation (activation) [160]. In addition, NELL-1 induced MAPK activity is accompanied by activation of phosphate transporters Pit1 and Pit2 to increase pre-osteoblast mineralization [162]. NELL-1 induction of Wnt signaling has been observed in both osteoblastic and osteoclastic cell types and is associated with its proosteogenic and antiosteoclastic effects [161]. The activation of HH signaling by NELL-1 has thus far been observed in preadipocytes only [42].

Recent data has shown that NELL-1 also exerts antiadipogenic effects [41]. These effects were found both in the preadipocyte cell line 3T3-L1 cells, as well as primary adipose-derived MSC (ASC) [41]. This was observed both in adipocyte specific gene expression and intracellular lipid accumulation. Recent *in vivo* studies have confirmed the antiadipogenic effects of NELL-1, in which direct intramedullary injection of NELL-1 reduced intramarrow adipocytes in a senile rat model [163]. This antiadipogenic effects of NELL-1 in preadipocytes is associated with activation of HH signaling, including HH signaling markers *Ihh, Gli1,* and *Ptc1*. Further studies found that coapplication of NELL-1 with cyclopamine, an antagonist for Smoothened, completely reversed or blunted the proosteogenic effects of NELL-1 [42]. Thus, NELL-1 is an osteoinductive cytokine with concomitant antiadipogenic properties. These effects may be through activation/intersection with MAPK, Wnt, and HH signaling.

## 8. Control of Adipogenesis and Osteogenesis by BMP Signaling

Bone morphogenetic proteins (BMPs), members of transforming growth factor-*β* (TGF-*β*) superfamily, are extracellular cytokines originally isolated from bone extract and found to induce of ectopic chondrogenesis and osteogenesis [164]. BMPs are responsible for numerous cell regulatory processes, including the differentiation and patterning of bone and cartilage [165]. Over 20 different BMPs have been identified, of which BMP-2, -4, -7, -9, and -13 are most commonly studied in the context of MSC differentiation [45, 166]. Both recombinant BMP-2 and -7 are approved by the FDA for the regeneration of bone in spinal fusion surgery and commonly used off-label for other orthopaedic applications [167, 168].

BMPs produce their effects through interaction with two serine-threonine kinase cell surface BMP receptors (BMPRs). Type II BMPRs initiate signaling upon binding to a BMP ligand, following which recruitment, phosphorylation, and activation of type I BMPRs occurs [165, 169, 170]. While there are several different type I BMPRs, only a few are involved in MSC differentiation, including BMPR-IA and BMPR-IB [47]. Several downstream BMP signaling elements exist, including Smad1/5/8, MAP Kinase, and c-Jun N-terminal kinase (JNK) signaling pathways, which are phosphorylated and thereby activated [47, 84, 171]. Of these, Smad1/5/8 signaling transduction is the most pertinent to MSC differentiation, as it is principally through the Smad-protein complexes that transcriptional regulation of adipogenic and osteogenic programming is regulated [165, 169, 170] (see [172] for a more detailed review of BMP signaling transduction).

BMP induced adipogenesis involves both Smad1/5/8 and MAPK activation [173]. BMP induced Smad1/5/8 signaling activates PPAR*γ* via zinc finger transcription factor Schnurri-2 and C/EBP*α*, which exhibit synergistic, adipogenic effects [33, 174]. Accordingly, a Smad antagonist such as Smad6 reduces both PPAR*γ* signaling and BMP-associated adipogenesis [173]. Similar to Smad1/5/8 signaling, BMP induced activation of MAPK signaling is associated with PPAR*γ* activation and adipogenic differentiation [173]. Conversely, disruption of MAPK signaling also inhibits both PPAR*γ* expression and BMP-associated adipogenesis [173]. Investigators have identified BMP signaling activity at the earliest stages of MSC adipogenesis [175, 176]. When MSC are forced into a preadipocyte cell lineage via exposure to 5-azacytidine, a potent inhibitor of DNA methylation, BMP-4 expression increases [175, 176]. BMP-4 has also been shown to have significance in brown adipose tissue, which prioritizes heat production over energy storage [177, 178]. Forced expression of BMP-4 in white adipocytes induces a brown adipocyte phenotype, including increased energy expenditure and insulin sensitivity [179]. Moreover, once MSC have been forced into preadipocyte cells, BMP-4 overexpression is sufficient to induce commitment to adipocyte lineage differentiation [45, 175, 180].

BMP signaling is one of the central signaling pathways involved in the induction of osteogenic differentiation and regulation of bone formation. Multiple murine studies involving genetically modified BMP ligands, BMP receptors, and BMP inhibitors demonstrate a critical role for BMP signaling in bone formation [181–184]. For example, transgenic mice with modified BMPR-IA receptors exhibit low bone mass and irregular calcification [181]. Inhibitors of BMP signaling, such as Noggin and Gremlin, impair bone formation when overexpressed [179, 185, 186]. In general, BMP induced osteogenesis utilizes both autocrine and paracrine pathways [187, 188] and works in conjunction with Osterix via both Runx2 dependent and independent pathways. BMP receptor activation in osteogenesis, as in adipogenesis, involves both Smad1/5/8 and MAPK downstream signaling activation. While 31 different BMP ligands are identified to date, only several actually promote MSC osteogenic differentiation [189]. Specifically, BMP-2, -4, -6, -7, and -9 have been shown to promote osteogenic commitment, as well as terminal osteogenic differentiation in MSC [45, 46]. BMP-2, the most commonly studied BMP ligand, induces MSC osteogenesis both *in vitro* and *in vivo* [190–197]. Furthermore, investigators have found that short-term BMP-2 treatment is both necessary and sufficient for osteogenic commitment in the C3H10T1/2 cell line [198]. It is important to note that murine-derived MSC in general show a robust osteogenic response to BMP signaling, whereas human MSC show a more variable response. For example, several studies evaluating BMP-2, -4, or -7 in human MSC did not observe reliably increased osteogenic differentiation [199]. Further investigation has suggested that higher expression of the BMP antagonist Noggin may underlie the variable response of human MSC to BMP-induced osteogenesis [200, 201].

The precise determinants that govern BMP signaling induced adipogenesis versus osteogenesis in MSC are not well understood. Two variables that may determine the effects of BMP on MSC differentiation have been observed: dosage and receptor type. In terms of dosage, lower concentrations of BMP-2 have been shown to directs towards adipocyte formation, while higher concentrations favor osteogenic differentiation in C3H10T1/2 [48]. However, these effects of dosage may be ligand- and cell-type dependent. In terms of receptor type, signaling through BMPR-IA in general induces adipogenic effects, while signaling via BMPR-1B induces osteogenic effects. For example, expression of constitutively active BMPR-IA induces adipogenic differentiation, while overexpression of inactive BMPR-IA inhibits adipogenic differentiation [47]. The converse effects were obtained by manipulation of BMPR-IB expression. Namely, constitutive BMPR-IB activation induces osteogenic differentiation while inactive BMPR-IB inhibited osteogenic differentiation [47]. However, conflicting data does exist regarding the specificity of BMPRs for lineage differentiation. For example, osteoblast-selective interference of BMPR-IA demonstrated anti-osteogenic effects including irregular calcification and decreased bone mass [181]. Thus, BMP receptor type and dosage are two known variables that have effect on MSC lineage determination, although no global rule applies [202].

## 9. Control of Adipogenesis and Osteogenesis by IGF Signaling

Discovered over fifty years ago, insulin-like growth factor-I (IGF-I) was originally identified as a soluble factor with insulin-like properties and induced by a growth hormone. Since then, we have developed a better understanding of this cytokine, especially in regard to its contribution towards bone formation and remodeling [203, 204] and adipogenesis. As a peptide hormone that acts in an endocrine, paracrine, and autocrine manner [205], IGF-1 primarily elicits effects via the IGF-I receptor (IGF1R) and IGF-binding proteins (IGFBPs) 1–6 [206]. While IGF-1 is primarily concentrated in the liver, it can be found systemically and is present in most peripheral tissues, including bone [204, 206, 207]. The functions of IGF-1 in bone have been well documented.

IGF-1 produces its effect by inducing several intracellular signaling pathways. IGF-1 first binds to the IGF-1 receptor, which autophosphorylates the receptor intracellularly at the kinase domain. With the receptor now activated, various protein substrates are consequently activated, including insulin receptor substrate-1 (IRS-1) and Src homology and collagen protein (SHC) [206]. IRS-1 goes on to activate the phosphoinositol 3-kinase (PI3-K), 3-PI-dependent kinase- (PDK-1), and Akt pathways, while SHC is responsible for activating the Ras/Raf/mitogen-activated protein (MAP) kinase pathways [207]. IRS-1 elicits its effect through interaction with and activation of PI3K, thereby catalyzing the phosphorylation of PIP2 to PIP3. The elevated levels of PIP3 consequently activate PDK-1 and Akt [208]. Activation of PI3K, PDK-1, and Akt has been shown to be important in skeletal growth [208, 209]. In fact, knockout Akt1/Akt2 mice demonstrate significantly impaired bone development and skeletal growth [208]. Meanwhile, SHC, which forms a complex with Grb2 and SOC, is responsible for increasing cell proliferation through activation of the Ras/Raf-1/MAPK pathway [206].

During bone remodeling, IGF-1 is released from the bone matrix to stimulate MSC osteoblastogenesis via activation of mammalian target of rapamycin (mTOR). This allows for the maintenance of both bone structure and mass, both of which were downregulated in mice with knockout of IGF-1 receptors in pre-osteoblastic cells [210]. Similarly, mice with deleted IGF-1 receptors in osteoclasts exhibit increased bone formation from decreased osteoclast formation [211]. Interestingly, IGF binding protein 3 is also a corequisite for IGF-1 in the bone matrix to stimulate new bone formation in rats [210]. Interestingly, while IGF binding protein 5 has exhibited proosteogenic properties in several studies, it also demonstrates inhibition of bone formation through impairing IGF-induced osteoblastogenesis [212]. Additionally, in serum-deprived conditions, MSC were shown to proliferate in response to IGF-1 [213]. Upstream, serum response factor (SRF) is found to regulate both IGF-1 and Runx2 signaling to control bone formation. In mice with conditional deletion of SRF in osteoblasts, Runx2 transactivity was restored via overexpression of SRF. SRF then plays an important role for IGF-1-induced osteoblastogenesis and mineralization through regulation of IGF-1 expression and Runx2 transactivity [214]. Collectively, these studies confirm the importance of IGF-1, its receptor, and respective binding protein for osteogenic differentiation and bone remodeling.

Combination of IGF-1 with various other growth factors provides additional insight on the mechanism of bone formation by IGF-1. For example, the addition of PDGF with IGF has been demonstrated to be more efficacious than either alone in terms of osteogenic induction in ASC [215]. Likewise, the combination of IGF-1 with AMD3100, an antagonist of chemokine receptor of CxCR4, showed significant augmentation of bone growth in segmental fracture murine models, associated with facilitation by the Akt/PI3K, MEK1/2-Erk1/2, and Smad2/3 signaling pathways [216]. In a distraction osteogenesis sheep model, application of both IGF-1 and TGF-*β*1 led to accelerated bone healing [217]. Another study found that growth hormone (GH) could increase to compensate for IGF-1 deficiency in mice to protect against inhibition of bone modeling during growth [218]. PTH is also known to stimulate both osteoblast and osteoclast function [211], with a role in modulating IGF-1 signaling through mechanisms involving IHH and ephrins [219]. Furthermore, there is a potential crosstalk between IGF-1 signaling and the integrin mechanosensing pathways, as evidenced by the failure of skeletal unloading to aid in bone growth despite IGF-1 infusion [219].

Interestingly, IGF-1 has been found to promote both adipogenic and osteogenic differentiation. For example, IGF-1 induces cell division of adipocyte precursor cells [220]. In addition, IGF receptors are involved in promoting adipogenesis through induction of advanced glycation end products (AGEs). AGEs activate both NAD(P)H oxidase and Src, which ultimately leads to the phosphorylation/activation of both IGF-1 receptor and Akt downstream in 3T3-L1 preadipocyte cells [221]. Further, Akt1/Akt2 knockout mice demonstrate impaired adipogenesis [208]. In fact, it has been shown that both Akt1 and Akt2 are necessary to induce PPAR*γ*, the key regulator for adipogenesis. Thus, a critical threshold of Akt activity, as regulated by IGF-1, contributes to the maintenance of cell proliferation, growth, and adipogenic differentiation [208].

## 10. Discussion

Numerous signaling pathways induce the adipogenic and/or osteogenic differentiation of MSC, not all of which were covered in this review. The majority of signaling pathways ultimately converge downstream affecting PPAR*γ* or Runx2 expression, transcriptional activity, or both. Although the mechanisms have not been fully discerned, many of these growth factors tend to elicit an “inverse relationship” between adipogenic and osteogenic differentiation. As discussed, Wnt, HH, and NELL-1 signaling follow this pattern, exhibiting proosteogenic/antiadipogenic effects [222]. Other well-studied signaling pathways further support this inverse relationship, including fibroblast growth factor-2 (FGF-2) [223], TGF-*β*1 [69, 224], and Notch signaling pathway [225], to name a few. Likewise, other transcription factors besides Runx2 demonstrate a proosteogenic, antiadipocytic relationship, one example being the recently described transcriptional activator TAZ (transcriptional activator with PDZ binding motif) [226]. However, there are a few exceptions to this pattern. For example, both IGF and BMP signaling have pleotropic, proosteogenic and proadipocytic properties [198, 227–229]. In summary, an inverse relationship exists between adipogenic and osteogenic lineage differentiation in MSC governed by diverse signaling pathways. The understanding of this relationship has far-reaching implications for the understanding of human health and treatment of human disease.
